# Transactions of the Medical Association of Southern Central New York, at the Annual Meeting, Held at Elmira, June, 1850

**Published:** 1850-10

**Authors:** 


*Transactions of the Medical Association of Southern Central New York, at the Annual Meeting, held at Elmira, June,* 1850.

A copy of the above publication was received some time since, and would have been noticed ere this, but for the pressure of other matters. We have had occasion before to advert to the success attending the organization of the Profession in the Southern central counties of this State. The Transactions for the present year betoken increased zeal on the part of those who have originated the enterprise. The plan pursued by this association is an excellent one, viz., to appoint a committee for each county to report on the endemical and epidemical diseases of the county during the year. If local associations existed in all parts of the country, and this plan were to be generally adopted, the materials for a comprehensive history of the diseases of successive years would be supplied—and in some such way only can this desirable end be attained. The American Medical Association appoints annually a committee to report on the Epidemics, etc., of the country, but how are the data for such a report to be obtained? We can see no mode so effectual, as the plan just referred to; and this furnishes not the least important of the numerous inducements to form and support local societies for scientific objects.

The Transactions of the Medical Association of Southern Central New York for the present year, make a pamphlet of eighty octavo pages. They consist, *first*, of an address by the President, Pelatiah B. Brooks, M. D., on the diseases of the kidneys and urinary organs; *second*, Report on the endemics and epidemics of Cortland county, by Frederick Hyde, M. D., chairman, embracing communications from Dr. H. P. Eels, Drs. Smith and Woodward, Dr. G. W. Bradford, and Dr. Caleb Green, all of whom were associate members of the committee of which Dr. Hyde was chairman; *third*, Report on the endemics and epidemics of Chemung county, by Wm. Woodward, M. D., chairman; *fourth*, Report on the endemics and epidemics of Tioga county, by Dr. H. N. Eastman, chairman. To the foregoing papers are appended a brief sketch of the life of the late Dr. Rowland Wilcox, by T. H. Squire, M. D.; and the proceedings of the annual meeting of the Association.

As a whole, the productions just enumerated are highly creditable to their authors, and to the Association. Their circulation will, we hope, incite members of the Profession in other sections of the country to unite for similar objects.

